# Supplementary material for: Detection and Characterization of Visceral Anisakid Nematodes in Blue Whiting from Portuguese Waters
Source: Foods. 2024 Nov 26;13(23):3802. doi: 10.3390/foods13233802 (PMC11640072; doi:10.3390/foods13233802)
Supplement: Supplementary file 1 [file foods-13-03802-s001.zip › foods-3306585-supplementary.pdf]

Fish label	Sample ID	Anisakid species	Infection with different species
1	F01.1	*Anisakis simplex*	No
	F01.2	*Anisakis simplex*	
2	F02.1	*Anisakis simplex*	Yes
	F02.2	*Anisakis pegreffii*	
3	F03.1	*Anisakis simplex*	No
	F03.2	*Anisakis simplex*	
4	F1.1	*Anisakis simplex*	No
	F1.2	*Anisakis simplex*	
5	F2.1	*Anisakis simplex*	Yes
	F2.2	*Hysterothylacium aduncum*	
6	F3.1	*Anisakis simplex*	Yes
	F3.2	*Anisakis pegreffii*	
7	F4.1	*Anisakis simplex*	Yes
	F4.2	*Hysterothylacium aduncum*	
8	F5.1	*Anisakis simplex*	Yes
	F5.2	*Hysterothylacium aduncum*	
9	F6.1	*Anisakis simplex*	Yes
	F6.2	*Anisakis pegreffii*	
10	F7.1	*Anisakis simplex*	No
	F7.2	*Anisakis simplex*	
11	F12.1	*Anisakis pegreffii*	Yes
	F8.2	*Hysterothylacium aduncum*	
12	F10.2	*Anisakis pegreffii*	NA
13	F13.1	*Anisakis pegreffii*	NA
14	F14.1	*Anisakis simplex*	Yes
	F14.2	*Anisakis pegreffii*	
15	F15.1	*Hysterothylacium aduncum*	No
	F15.2	*Hysterothylacium aduncum*	
16	F16.1	*Anisakis simplex*	No
	F16.2	*Anisakis simplex*	
17	F17.1	*Anisakis simplex*	No
	F17.2	*Anisakis simplex*	
18	F18.1	*Anisakis simplex*	No
	F18.2	*Anisakis simplex*	
19	F19.1	*Anisakis simplex*	NA
20	F20.1	*Anisakis simplex*	No
	F20.2	*Anisakis simplex*	
21	F24.1	*Anisakis simplex*	No
	F24.2	*Anisakis simplex*	
22	F25.1	*Hysterothylacium aduncum*	Yes
	F25.2	*Anisakis simplex*	
23	F26.1	*Anisakis simplex*	No

	F46.2	*Anisakis simplex*	
24	F27.1	*Anisakis simplex*	Yes
	F27.2	*Hysterothylacium aduncum*	
25	F28.1	*Anisakis pegreffii*	No
	F28.2	*Anisakis pegreffii*	
26	F29.1	*Anisakis simplex*	No
	F29.2	*Anisakis simplex*	
27	F30.1	*Anisakis pegreffii*	Yes
	F30.2	*Anisakis simplex*	
28	F31.1	*Anisakis pegreffii*	Yes
	F31.2	*Anisakis simplex*	
29	F32.1	*Anisakis simplex*	No
	F33.2	*Anisakis simplex*	
30	F34.1	*Hysterothylacium aduncum*	Yes
	F34.2	*Anisakis pegreffii*	
31	F36.1	*Anisakis simplex*	No
	F35.2	*Anisakis simplex*	
32	F38.1	*Anisakis simplex*	No
	F38.2	*Anisakis simplex*	
33	F40.1	*Anisakis simplex*	Yes
	F40.2	*Anisakis pegreffii*	
34	F43.1	*Anisakis simplex*	Yes
	F43.2	*Anisakis pegreffii*	
35	F45.1	*Anisakis simplex*	No
	F45.2	*Anisakis simplex*	
36	F48.1	*Anisakis simplex*	No
	F48.2	*Anisakis simplex*	
37	F49.1	*Anisakis simplex*	Yes
	F63.2	*Hysterothylacium aduncum*	
38	F50.1	*Anisakis simplex*	Yes
	F50.2	*Anisakis pegreffii*	
39	F51.1	*Anisakis simplex*	Yes
	F51.2	*Anisakis pegreffii*	
40	F53.1	*Anisakis simplex*	No
	F53.2	*Anisakis simplex*	
41	F54.1	*Anisakis simplex*	No
	F54.2	*Anisakis simplex*	
42	F56.1	*Anisakis simplex*	No
	F56.2	*Anisakis simplex*	
43	F65.1	*Anisakis simplex*	No
	F65.2	*Anisakis simplex*	
44	F66.1	*Anisakis simplex*	No
	F66.2	*Anisakis simplex*	
45	F68.1	*Anisakis simplex*	Yes
	F70.2	*Hysterothylacium aduncum*	
46	F69.1	*Anisakis pegreffii*	Yes

	F69.2	*Hysterothylacium aduncum*	
47	F72.1	*Anisakis simplex*	NA
48	F76.1	*Anisakis simplex*	NA
49	F79.1	*Anisakis simplex*	Yes
	F84.2	*Hysterothylacium aduncum*	
50	F41.2	*Anisakis simplex*	NA

Table S1 : Species identification of the largest and smallest Anisakid larvae collected from each blue whiting fish (*Micromesistious poutassou)* and co-infection rates. 'Yes' indicates co-infection, where different Anisakid species were present in the same fish sample, 'No' indicates that both the largest and smallest larvae belonged to the same species, signifying no co-infection, and 'NA' denotes that the data collected were insufficient to assess the presence of co-infection in the fish sample.

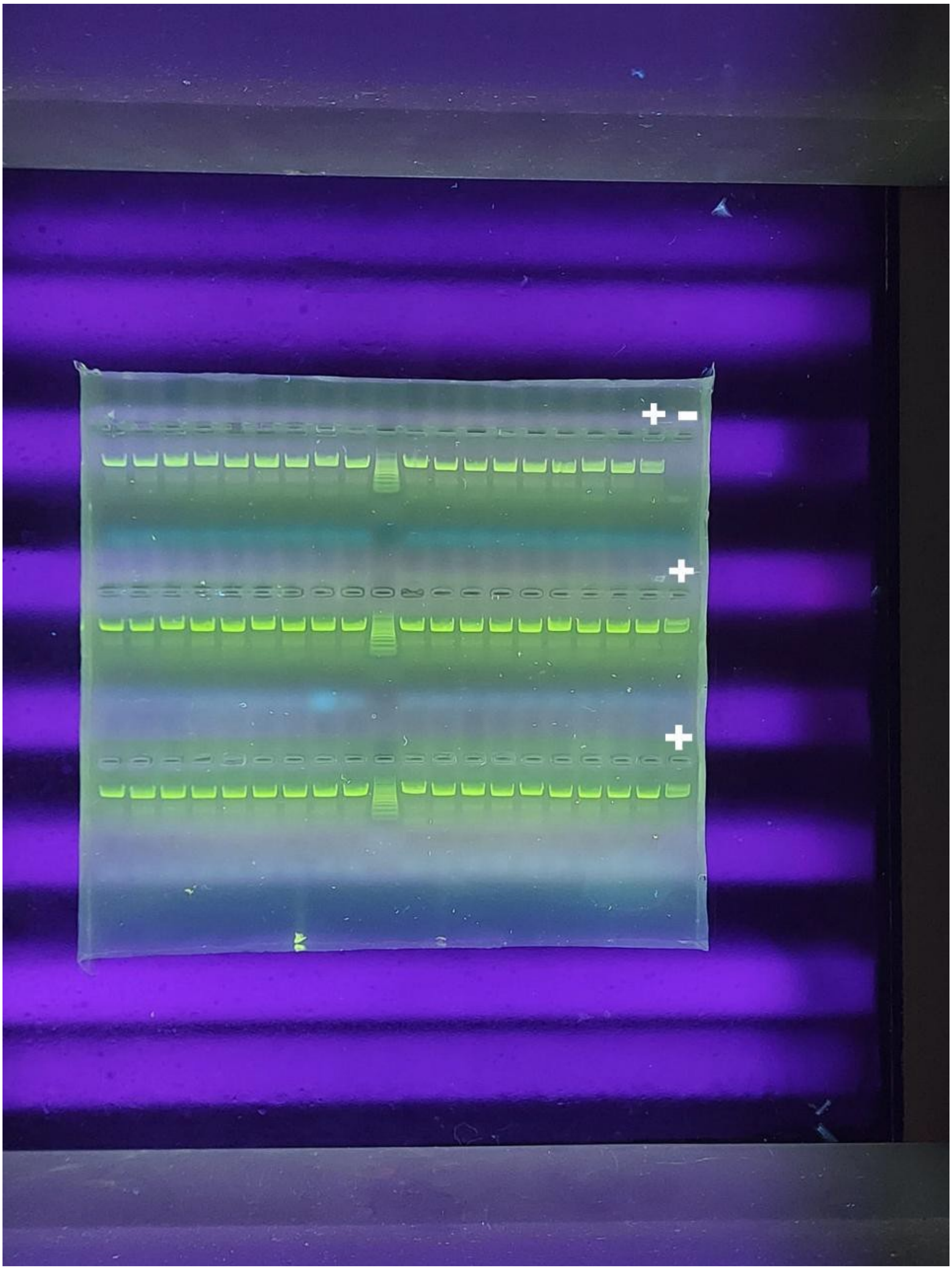

Figure S1: Representative electrophoresis gel image displaying amplification results for the 950 bp ITS region, with clear positives for Anisakid species. The '+' indicates the positive control, which successfully amplified, confirming the assay's specificity, while the '–' denotes the negative control, which did not amplify, validating the absence of contamination
